# Energy-Trapping Characteristics of Lateral Field Excited GdCOB Crystal Bulk Acoustic Wave Devices Based on Stepped Electrodes

**DOI:** 10.3390/mi14122162

**Published:** 2023-11-27

**Authors:** Bowei Wu, Pengfei Kang, Tingfeng Ma, Yuming Yao, Ning Gan, Peng Li, Zhenghua Qian, Iren Kuznetsova, Ilya Nedospasov, Wenhui Hu

**Affiliations:** 1 School of Mechanical Engineering and Mechanics, Ningbo University, Ningbo 315211, China; 2211090034@nbu.edu.cn (B.W.); 2211090019@nbu.edu.cn (P.K.); 2 Keli Sensing Technology (Ningbo) Co., Ltd., Ningbo 315033, China; keliyym@kelichina.com (Y.Y.); 15957859248@163.com (W.H.); 3 School of Material Science and Chemical Engineering, Ningbo University, Ningbo 315211, China; ganning@nbu.edu.cn; 4 The State Key Laboratory of Mechanics and Control of Mechanical Structures, College of Aerospace Engineering, Nanjing University of Aeronautics and Astronautics, Nanjing 210016, China; lipeng_mech@nuaa.edu.cn (P.L.); qianzh@nuaa.edu.cn (Z.Q.); 5 Kotelnikov Institute of Radio Engineering and Electronics of RAS, 125009 Moscow, Russia; kuziren@yandex.ru (I.K.); ianedospasov@mail.ru (I.N.)

**Keywords:** lateral field excitation, stepped electrodes, monoclinic crystals, energy trapping

## Abstract

In this work, high-frequency forced vibrations of lateral field excitation (LFE) devices with stepped electrodes based on monoclinic crystals GdCOB are modeled, and the influence laws of the device parameters (the step number, size, and thickness of the stepped electrodes) on the energy-trapping effects of the device are revealed. The results show that the step number has a significant effect on the energy-trapping effect of the device: with the increase in the step number, the stronger energy-trapping effect of the device can be obtained; with the increase in the thickness difference of two layers of electrodes, the energy-trapping effect of the device becomes stronger; with the increase in the difference of the electrode radius, the energy-trapping effect of the device is enhanced gradually. The results of this work can provide an important theoretical basis for the design of stepped-electrode LFE resonators and sensors with high-quality factors based on monoclinic crystals.

## 1. Introduction

Piezoelectric bulk acoustic wave devices are widely used in gas-phase and liquid-phase sensing due to their high accuracy, stability, and consistency [1,2,3,4,5,6,7,8]. Conventional piezoelectric bulk acoustic wave devices rely on a thickness-field-excitation (TFE) mode, in which electrodes are plated on the top and bottom surfaces of a crystal plate, and the resulting effective electric field is in the direction of the thickness of the crystal plate [9,10,11,12]. Previous research on piezoelectric devices operating in has shown some problems: (1) it is not easy to package the device after it is fabricated; (2) when used in a liquid phase or other corrosive environments, the electrodes of the device are exposed to the outer environment, which accelerates the corrosion of the electrodes and shortens the service life of the device [13]. The lateral field excitation (LFE) mode has been proposed in recent years, for which two electrodes are located on the surface of a crystal plate; thus, the direction of the generated effective electric field is parallel to the surface of the crystal plate [14,15,16,17]. The LFE devices not only solve the problems of packaging and the short service life of TFE devices but also bring some advantages, such as the following: (1) The resonance damping of the device under the lateral field is smaller, so that the energy-trapping effect of the device is better and the corresponding sensing sensitivity is higher. (2) By changing the angle of the lateral electric field, the electromechanical coupling coefficient of the LFE device can be improved, and the frequency stability of the device can be obviously enhanced. 

Because the density of the electrode material is usually much greater than that of the crystal material, the thickness-shear vibration energy in the partially electrode piezoelectric crystal plate is limited to the area covered by the electrodes, and the phenomenon of exponentially attenuating the vibration amplitude with the increase in distance from the electrode is called energy trapping [18,19,20,21,22]. The energy-trapping effect directly determines the resonance quality factor of piezoelectric bulk acoustic wave devices, which is closely related to the stability of the devices and is particularly important for bulk acoustic wave sensing. The methods to obtain good energy trapping mainly include optimizing electrode shape, but the improvement in the energy-trapping effect is very limited [23]. In recent years, stepped electrodes have been proposed to improve the energy-trapping effect of devices, and the crystal plate with stepped electrodes excited by TFE has been proven to have a better energy-trapping effect [24]. However, the existing studies on bulk acoustic wave devices with stepped electrodes mainly focus on the TFE mode, and for the LFE mode, due to the complex electric field and displacement distribution, the influences of the stepped electrode on the energy-trapping effect are not clear, and the corresponding design criteria are lacking.

In this work, using the Mindlin plate theory, we established a theoretical model of high-frequency vibrations of LFE piezoelectric bulk acoustic wave devices with stepped electrodes based on monoclinic GdCOB crystals, which have more stable electromechanical properties over the temperature range of 20 to 1000 °C, low dielectric loss [25], and analyzed the influences of stepped electrodes on the energy-trapping characteristics of LFE bulk acoustic wave devices.

## 2. Governing Equation

**Figure 1:**
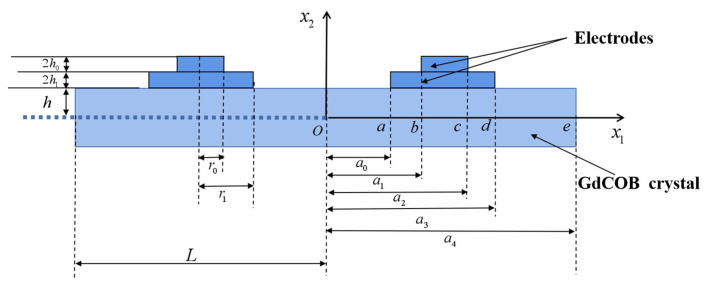
The GdCOB crystal plate with stepped electrodes under lateral field excitation.

The schematic diagram of the LFE device with a two-layer stepped electrode based on GdCOB crystals is shown in Figure 1. The thickness of the crystal plate is 2*h*, the length is 2*L*, and the density is ρ. The thickness of the upper and lower layers of electrodes is $2h_{0}$ and $2h_{1}$, respectively, and the density is $\rho^{\prime }$. The normal direction of the crystal plate is along the $x_{2}$ axis, where $x_{3}$ axis is determined from $x_{1}$ and $x_{2}$ axes by the right-hand rule, the crystal plate is symmetric about $x_{1}$ = 0, and unbounded in the $x_{3}$ orientation.

The $a<\left|x_{1}\right|<b$,$\;c<\left|x_{1}\right|<d$ regions are covered with single-layer electrodes, the $b<\left|x_{1}\right|<c$ region is covered by double-layer electrodes, and the device electrodes exert an alternating voltage of ±Vexp(iωt), which generates an electric field of $E_{1}\left(x_{1},t\right)$ in the $\left|x_{1}\right|<a$ region.

According to the Mindlin plate theory, the following assumptions are made for the displacements and potentials in the non-electrode region of the plate [26,27]:

(1) $$u_{3}≅x_{2}u_{3}^{\left(1\right)}(x_{1},t),\;u_{2}≅u_{2}^{\left(0\right)}(x_{1},t),\phi ≅\phi^{\left(1\right)}(x_{1},t),\;u_{1}≅x_{2}u_{1}^{\left(1\right)}(x_{1},t),$$

where $u_{1}^{\left(1\right)}(x_{1},t)$ is the thickness shear strain, $u_{3}^{\left(1\right)}(x_{1},t)$ is the thickness-twist strain, $u_{2}^{\left(0\right)}(x_{1},t)$ is the bending strain, and $\phi^{\left(1\right)}$ is the electric potential. Considering only the wave propagation in the x-direction, the partial derivative of $x_{3}$ in the equation of motion is set to 0.

For LFE bulk acoustic wave devices based on monoclinic crystals, the motion equations for the non-electrode region are as follows [28]:

(2) $$\begin{array}{l} k_{1}k_{3}C_{64}u_{3,1}^{\left(1\right)}+k_{1}^{2}C_{66}(u_{2,11}^{\left(0\right)}+u_{1,1}^{\left(1\right)})+k_{1}e_{26}\phi_{,1}^{\left(1\right)}=\rho {\ddot{u}}_{2}^{\left(0\right)},\\ C_{51}u_{1,11}^{\left(1\right)}+C_{55}u_{3,11}^{\left(1\right)}+e_{15}\phi_{,11}^{\left(1\right)}-\frac{3}{h^{2}}[k_{3}^{2}C_{44}u_{3}^{\left(1\right)}+k_{1}k_{3}C_{46}(u_{2,1}^{\left(0\right)}+u_{1}^{\left(1\right)})+k_{3}e_{24}\phi^{\left(1\right)}]=\rho {\ddot{u}}_{3}^{\left(1\right)},\\ C_{11}u_{1,11}^{\left(1\right)}+C_{15}u_{3,11}^{\left(1\right)}+e_{11}\phi_{,11}^{\left(1\right)}-\frac{3}{h^{2}}[k_{1}k_{3}C_{64}u_{3}^{\left(1\right)}+k_{1}^{2}C_{66}(u_{2,1}^{\left(0\right)}+u_{1}^{\left(1\right)})+k_{1}e_{26}\phi^{\left(1\right)}]=\rho {\ddot{u}}_{1}^{\left(1\right)},\\ e_{11}u_{1,11}^{\left(1\right)}+\varepsilon_{11}\phi_{,11}^{\left(1\right)}+e_{15}u_{3,11}^{\left(1\right)}-\frac{3}{h^{2}}[-\varepsilon_{22}\phi^{\left(1\right)}+k_{3}e_{24}u_{3}^{\left(1\right)}+k_{1}e_{26}(u_{2,1}^{\left(0\right)}+u_{1}^{\left(1\right)})]=0, \end{array}$$

where $k_{1}=\sqrt{\frac{\pi^{2}}{12}}$$k_{3}=\sqrt{\frac{\pi^{2}\cdot C_{3}}{12\cdot C_{44}}}$, $C_{3}=(C_{22}+C_{44}-\sqrt{{\left(C_{22}-C_{44}\right)}^{2}+4\cdot C_{24}{}^{2}})/2$.

For the electrode region, since the stiffness of the electrode has a negligible effect on the device when the device operating frequency is below 100 MHz, the electrodes on the crystal plate can be assumed to be rigidly adhered, and the electrode mass is the only influencing factor [29]. Thus, the concept of mass ratio is introduced:

(3) $$R=\frac{\rho^{\prime }h^{\prime }}{\rho h}<<1.$$

In Equation (3), h and $h^{\prime }$ correspond to half of the crystal and electrode thicknesses, respectively, and the value of the mass ratio *R* is much less than 1. The governing equations for the electrode region are as follows:

(4) $$\begin{array}{l} k_{1}k_{3}C_{64}u_{3,1}^{\left(1\right)}+k_{1}^{2}C_{66}(u_{2,11}^{\left(0\right)}+u_{1,1}^{\left(1\right)})+{\bar{k}}_{1}e_{26}\phi_{,1}^{\left(1\right)}=\rho (1+R){\ddot{u}}_{2}^{\left(0\right)},\\ C_{51}u_{1,11}^{\left(1\right)}+C_{55}u_{3,11}^{\left(1\right)}-\frac{3}{h^{2}}[k_{3}^{2}C_{44}u_{3}^{\left(1\right)}+k_{1}k_{3}C_{46}(u_{2,1}^{\left(0\right)}+u_{1}^{\left(1\right)})+{\bar{k}}_{3}e_{24}\phi^{\left(1\right)}]=\rho (1+3R){\ddot{u}}_{3}^{\left(1\right)},\\ C_{11}u_{1,11}^{\left(1\right)}+C_{15}u_{3,11}^{\left(1\right)}-\frac{3}{h^{2}}[k_{1}k_{3}C_{64}u_{3}^{\left(1\right)}+k_{1}^{2}C_{66}(u_{2,1}^{\left(0\right)}+u_{1}^{\left(1\right)})+{\bar{k}}_{1}e_{26}\phi^{\left(1\right)}]=\rho (1+3R){\ddot{u}}_{1}^{\left(1\right)},\\ e_{11}u_{1,11}^{\left(1\right)}-\varepsilon_{11}\phi_{,11}^{\left(1\right)}+e_{15}u_{3,11}^{\left(1\right)}-\frac{3}{h^{2}}[-\varepsilon_{22}\phi^{\left(1\right)}+{\bar{k}}_{3}e_{24}u_{3}^{\left(1\right)}+{\bar{k}}_{1}e_{26}(u_{2,1}^{\left(0\right)}+u_{1}^{\left(1\right)})]=0, \end{array}$$

where ${\bar{k}}_{1}^{2}=k_{1}^{2}(1+R)$,${\bar{k}}_{3}^{2}=k_{3}^{2}(1+R)$.

## 3. Forced Vibrations of Finite Crystal Plates

Since the plate is symmetric at about $x_{1}$ = 0 and an antisymmetric voltage is applied to the electrode plate, the electromechanical coupling field is also symmetric or antisymmetric at about $x_{1}$ = 0. Therefore, only half of the crystal plate is considered in this work.

### 3.1. Central Non-Electrode Area $\left(0<x_{1}<a\right)$

According to the standing wave assumption of the finite plate, the displacement and potential of the crystal plate under forced vibrations are given as

(5) $$\begin{array}{l} u_{2}^{\left(0\right)}=A_{1}\sin(\xi x_{1}-wt),\\ u_{3}^{\left(1\right)}=A_{2}\cos(\xi x_{1}-wt),\\ u_{1}^{\left(1\right)}=A_{3}\cos(\xi x_{1}-wt),\\ \phi^{\left(1\right)}=A_{4}\cos(\xi x_{1}-wt), \end{array}$$

where $A_{1}-A_{4}$ are constants to be determined. Substituting Equation (5) into Equation (2) yields a fourth-order linear equation on $A_{1}-A_{4}$. The determinant of the coefficient matrix must be zero for nontrivial solutions, which yields a fourth-order polynomial in terms of $\xi^{2}$. Solving this polynomial yields four roots, which can be expressed as ${\left(\xi^{\left(m\right)}\right)}^{2}$, where m = 1–4. Therefore, the corresponding solution of the linear equation with respect to the non-zero solution ξ(m) is $\beta_{p}^{\left(m\right)}$, where m = 1–3. $\beta_{p}^{\left(m\right)}$ is determined by the ratio of the amplitudes $A_{1}-A_{4}$. The following symmetric solutions can be built:

(6) $$\left\{\begin{array}{c} u_{2}^{\left(0\right)}\\ u_{3}^{\left(1\right)}\\ u_{1}^{\left(1\right)}\\ \phi^{\left(1\right)} \end{array}\right\}=\sum_{m=1}^{4}C^{\left(m\right)}\left\{\begin{array}{c} \beta_{1}^{\left(m\right)}\sin(\xi^{\left(m\right)}x_{1})\\ \beta_{2}^{\left(m\right)}\cos(\xi^{\left(m\right)}x_{1})\\ \beta_{3}^{\left(m\right)}\cos(\xi^{\left(m\right)}x_{1})\\ \beta_{4}^{\left(m\right)}\cos(\xi^{\left(m\right)}x_{1}) \end{array}\right\},$$

where $C^{\left(1\right)}-C^{\left(4\right)}$ are unknown constants.

### 3.2. Area Covered by a Single-Layer Electrode $\left(a<x_{1}<b,c<x_{1}<d\right)$

In the area covered by a single-layer electrode layer, the corresponding displacement and electrical-potential assumptions need to be added by a specific solution:

(7) $$\begin{array}{l} u_{2}^{\left(0\right)}=A_{1}e^{i\bar{\xi_{1}}x_{1}}e^{iwt},\\ u_{3}^{\left(1\right)}=A_{2}e^{i{\bar{\xi }}_{1}x_{1}}e^{iwt}+\tilde{u_{3}^{\left(1\right)}},\\ u_{1}^{\left(1\right)}=A_{3}e^{i{\bar{\xi }}_{1}x_{1}}e^{iwt}+\tilde{u_{1}^{\left(1\right)}},\\ \phi^{\left(1\right)}=A_{4}e^{i{\bar{\xi }}_{1}x_{1}}e^{iwt}+\tilde{\phi^{\left(1\right)}} \end{array}$$

Substituting Equation (7) into Equation (4) yields a fourth-order linear equation about $A_{1}-A_{4}$. $A_{1}-A_{4}$ has a non-zero solution, namely the coefficient matrix determinant is zero, which yields a quadratic equation of ${\bar{\xi }}^{2}{}_{1}$. Four pairs of non-zero solutions can be obtained by solving the equation, denoted as ${\bar{\xi }}_{1}{}^{\left(m\right)}$ (m = 1–8). With respect to the non-zero solution, the corresponding solution of the linear equation is ${\bar{\beta }}_{p}^{\left(m\right)}$, where m = 1–4. $\beta_{p}^{\left(m\right)}$ is determined by the ratio of the amplitudes *A*_1_ − *A*_4_. Then, the following solutions can be built:

(8) $$\left\{\begin{array}{c} u_{2}^{\left(0\right)}\\ u_{3}^{\left(1\right)}\\ u_{1}^{\left(1\right)}\\ \phi^{\left(1\right)} \end{array}\right\}=\sum_{m=1}^{8}{\bar{C_{1}}}^{\left(m\right)}\left\{\begin{array}{c} {\bar{\beta }}_{1}{}^{\left(m\right)}e^{i{\bar{\xi_{1}}}^{\left(m\right)}x_{1}}\\ {\bar{\beta }}_{2}{}^{\left(m\right)}e^{i{\bar{\xi_{1}}}^{\left(m\right)}x_{1}}\\ {\bar{\beta }}_{3}{}^{\left(m\right)}e^{i{\bar{\xi }}_{1}{}^{\left(m\right)}x_{1}}\\ {\bar{\beta }}_{4}{}^{\left(m\right)}e^{i{\bar{\xi }}_{1}{}^{\left(m\right)}x_{1}} \end{array}\right\}+\left\{\begin{array}{c} 0\\ B_{1}\\ B_{2}\\ B_{3} \end{array}\right\},$$

where ${\bar{C_{1}}}^{\left(1\right)}-{\bar{C_{1}}}^{\left(8\right)}$ are unknown constants, and the $B_{1},B_{2}$ and $B_{3}$ satisfy the following equations:

(9) $$\begin{array}{l} {}[\frac{-12\cdot {\left({\bar{k}}_{1}\right)}^{2}\cdot C_{66}}{\pi^{2}}+c_{66}\Omega^{2}\cdot (1+3R_{1})]B_{1}-\frac{12{\bar{k}}_{1}{\bar{k}}_{3}C_{64}}{\pi^{2}}B_{2}=\frac{12\cdot {\bar{k}}_{1}\cdot e_{26}\cdot V}{\pi^{2}L},\\ (\frac{-12{\bar{\cdot k}}_{1}\cdot {\bar{k}}_{3}\cdot C_{64}}{\pi^{2}})B_{1}+[\frac{-12{\left({\bar{k}}_{3}\right)}^{2}C_{44}}{\pi^{2}}+c_{66}\Omega^{2}\cdot (1+3R_{1})]B_{2}=\frac{12\cdot {\bar{k}}_{3}\cdot e_{24}\cdot V}{\pi^{2}L},\\ B_{3}=V/L. \end{array}$$

### 3.3. Area Covered by the Double-Layer Electrodes $\left(b<x_{1}<c\right)$

For the area covered by the double-layer electrodes, the fourth-order linear equation of $A_{1}-A_{4}$ can be obtained. Four pairs of non-zero solutions can be obtained by solving the equation, denoted as ${\left({\hat{\xi }}^{\left(m\right)}\right)}^{2}$ (m = 1–8); Now, the following general symmetric solutions can be built:

(10) $$\left\{\begin{array}{c} u_{2}^{\left(0\right)}\\ u_{3}^{\left(1\right)}\\ u_{1}^{\left(1\right)}\\ \phi^{\left(1\right)} \end{array}\right\}=\sum_{m=1}^{8}{\hat{C}}^{\left(m\right)}\left\{\begin{array}{c} {\hat{\beta }}_{1}{}^{\left(m\right)}e^{i{\hat{\xi }}^{\left(m\right)}x_{1}}\\ {\hat{\beta }}_{2}{}^{\left(m\right)}e^{i{\hat{\xi }}^{\left(m\right)}x_{1}}\\ {\hat{\beta }}_{3}{}^{\left(m\right)}e^{i{\hat{\xi }}^{\left(m\right)}x_{1}}\\ {\hat{\beta }}_{4}{}^{\left(m\right)}e^{i{\hat{\xi }}^{\left(m\right)}x_{1}} \end{array}\right\}+\left\{\begin{array}{c} 0\\ {\hat{B}}_{1}\\ {\hat{B}}_{2}\\ {\hat{B}}_{3} \end{array}\right\},$$

where ${\hat{C}}^{\left(1\right)}$–${\hat{C}}^{\left(8\right)}$ are unknown constants, and ${\hat{B}}_{1}$, ${\hat{B}}_{2}$ and ${\hat{B}}_{3}$ satisfy the following equations:

(11) $$\begin{array}{l} {}[\frac{-12\cdot {\left({\bar{k}}_{1}\right)}^{2}\cdot C_{66}}{\pi^{2}}+c_{66}\Omega^{2}\cdot (1+3R_{2})]{\hat{B}}_{1}-\frac{12{\bar{k}}_{1}{\bar{k}}_{3}C_{64}}{\pi^{2}}{\hat{B}}_{2}=\frac{12\cdot {\bar{k}}_{1}\cdot e_{26}\cdot V}{\pi^{2}L},\\ (\frac{-12{\bar{\cdot k}}_{1}\cdot {\bar{k}}_{3}\cdot C_{64}}{\pi^{2}}){\hat{B}}_{1}+[\frac{-12{\left({\bar{k}}_{3}\right)}^{2}C_{44}}{\pi^{2}}+c_{66}\Omega^{2}\cdot (1+3R_{2})]{\hat{B}}_{2}=\frac{12\cdot {\bar{k}}_{3}\cdot e_{24}\cdot V}{\pi^{2}L},\\ {\hat{B}}_{3}=V/L. \end{array}$$

### 3.4. External Non-Electrode Area $\left(d<x_{1}<e\right)$

For the external non-electrode area, the displacement and electrical potential are assumed as follows:

(12) $$\begin{array}{l} u_{2}^{\left(0\right)}=A_{1}e^{i\tilde{\xi }x_{1}}e^{iwt}\\ u_{3}^{\left(1\right)}=A_{2}e^{i\tilde{\xi }x_{1}}e^{iwt}\\ u_{1}^{\left(1\right)}=A_{3}e^{i\tilde{\xi }x_{1}}e^{iwt}\\ \phi^{\left(1\right)}=A_{4}e^{i\tilde{\xi }x_{1}}e^{iwt} \end{array}$$

Substituting Equation (12) into Equation (4) yields a fourth-order linear equation of $A_{1}-A_{4}$. For a non-zero solution, the coefficient matrix determinant is zero, which yields a polynomial equation of degree four of ${\tilde{\xi }}^{2}$. A fourth-order polynomial about $\xi^{2}$ is obtained. Solving this polynomial yields eight roots, which can be expressed as ${\left(\tilde{\xi }\right)}^{m}$ (*m* = 1–8). With respect to the non-zero solution ${\left(\tilde{\xi }\right)}^{m}$, the corresponding solution of the linear equation is $\beta_{p}^{\left(m\right)}$. $\beta_{p}^{\left(m\right)}$ is determined by the ratio of the amplitudes of $A_{1}\;-\;A_{4}$. Then, the following general symmetric solutions can be built:

(13) $$\left\{\begin{array}{c} u_{2}^{\left(0\right)}\\ u_{3}^{\left(1\right)}\\ u_{1}^{\left(1\right)}\\ \phi^{\left(1\right)} \end{array}\right\}=\sum_{m=1}^{8}{\tilde{C}}^{\left(m\right)}\left\{\begin{array}{c} {\tilde{\beta }}_{1}^{\left(m\right)}\sin({\tilde{\xi }}^{\left(m\right)}x_{1})\\ {\tilde{\beta }}_{2}^{\left(m\right)}\cos({\tilde{\xi }}^{\left(m\right)}x_{1})\\ {\tilde{\beta }}_{3}^{\left(m\right)}\cos({\tilde{\xi }}^{\left(m\right)}x_{1})\\ {\tilde{\beta }}_{4}^{\left(m\right)}\cos({\tilde{\xi }}^{\left(m\right)}x_{1}) \end{array}\right\},$$

where ${\tilde{C}}^{\left(1\right)}-{\tilde{C}}^{\left(8\right)}$ is the unknown constant, and ${\tilde{\beta }}_{4}^{\left(m\right)}=1$.

### 3.5. Boundary and Continuity Conditions

For the right half of the plate shown in Figure 1, the boundary and continuous conditions are shown below.

At $x_{1}=a$, the continuity conditions are

(14) $$\begin{array}{l} u_{2}^{\left(0\right)}(x_{1}=a^{-})=u_{2}^{\left(0\right)}(x_{1}=a^{+})\\ u_{3}^{\left(1\right)}(x_{1}=a^{-})=u_{3}^{\left(1\right)}(x_{1}=a^{+})\\ u_{1}^{\left(1\right)}(x_{1}=a^{-})=u_{1}^{\left(1\right)}(x_{1}=a^{+})\\ T_{6}^{\left(0\right)}(x_{1}=a^{-})=T_{6}^{\left(0\right)}(x_{1}=a^{+})\\ T_{5}^{\left(1\right)}(x_{1}=a^{-})=T_{5}^{\left(1\right)}(x_{1}=a^{+})\\ T_{1}^{\left(1\right)}(x_{1}=a^{-})=T_{1}^{\left(1\right)}(x_{1}=a^{+})\\ D_{1}^{\left(1\right)}(x_{1}=a^{-})=D_{1}^{\left(1\right)}(x_{1}=a^{+})\\ \phi^{\left(1\right)}(x_{1}=a^{-})=\phi^{\left(1\right)}(x_{1}=a^{+}) \end{array}$$

At $x_{1}=b$, the continuity conditions are

(15) $$\begin{array}{l} u_{2}^{\left(0\right)}(x_{1}=b^{-})=u_{2}^{\left(0\right)}(x_{1}=b^{+})\\ u_{3}^{\left(1\right)}(x_{1}=b^{-})=u_{3}^{\left(1\right)}(x_{1}=b^{+})\\ u_{1}^{\left(1\right)}(x_{1}=b^{-})=u_{1}^{\left(1\right)}(x_{1}=b^{+})\\ T_{6}^{\left(0\right)}(x_{1}=b^{-})=T_{6}^{\left(0\right)}(x_{1}=b^{+})\\ T_{5}^{\left(1\right)}(x_{1}=b^{-})=T_{5}^{\left(1\right)}(x_{1}=b^{+})\\ T_{1}^{\left(1\right)}(x_{1}=b^{-})=T_{1}^{\left(1\right)}(x_{1}=b^{+})\\ D_{1}^{\left(1\right)}(x_{1}=b^{-})=D_{1}^{\left(1\right)}(x_{1}=b^{+})\\ \phi^{\left(1\right)}(x_{1}=b^{-})=\phi^{\left(1\right)}(x_{1}=b^{+}) \end{array}$$

At $x_{1}=d$, the continuity conditions are

(16) $$\begin{array}{l} u_{2}^{\left(0\right)}(x_{1}=d^{-})=u_{2}^{\left(0\right)}(x_{1}=d^{+})\\ u_{3}^{\left(1\right)}(x_{1}=d^{-})=u_{3}^{\left(1\right)}(x_{1}=d^{+})\\ u_{1}^{\left(1\right)}(x_{1}=d^{-})=u_{1}^{\left(1\right)}(x_{1}=d^{+})\\ T_{6}^{\left(0\right)}(x_{1}=d^{-})=T_{6}^{\left(0\right)}(x_{1}=d^{+})\\ T_{5}^{\left(1\right)}(x_{1}=d^{-})=T_{5}^{\left(1\right)}(x_{1}=d^{+})\\ T_{1}^{\left(1\right)}(x_{1}=d^{-})=T_{1}^{\left(1\right)}(x_{1}=d^{+})\\ D_{1}^{\left(1\right)}(x_{1}=d^{-})=D_{1}^{\left(1\right)}(x_{1}=d^{+})\\ \phi^{\left(1\right)}(x_{1}=d^{-})=\phi^{\left(1\right)}(x_{1}=d^{+}) \end{array}$$

At $x_{1}=e$, the continuity conditions are:

(17) $$\begin{array}{l} T_{6}^{\left(0\right)}(x_{1}=e^{-})=0\\ T_{5}^{\left(1\right)}(x_{1}=e^{-})=0\\ T_{1}^{\left(1\right)}(x_{1}=e^{-})=0\\ D_{1}^{\left(1\right)}(x_{1}=e^{-})=0 \end{array}$$

The unknown constants $C^{\left(1\right)}-C^{\left(4\right)},$ ${\bar{C}}_{1}{}^{\left(1\right)}-{\bar{C}}_{1}{}^{\left(8\right)},$ ${\hat{C}}^{\left(1\right)}-{\hat{C}}^{\left(8\right)}$, and ${\tilde{C}}^{\left(1\right)}-{\tilde{C}}^{\left(8\right)}$ can be obtained by substituting Equations (6), (8), (10), and (13) into Equations (14)–(17), and the corresponding displacement solutions and electric potential solutions can be achieved. The charge $Q_{e}$ and dynamic capacitance C can be obtained as follows:

(18) $$\begin{array}{l} Q_{e}=-D_{1}^{\left(1\right)}(x=a)\cdot 2w,C=\frac{Q_{e}}{2V},\;\\ C_{0}=\frac{4\varepsilon_{11}hw}{2c}, \end{array}$$

where *C*_0_ is the static capacitance.

## 4. Mode Coupling Analysis

In this section, parameters of the resonators are set as 2h=0.06138 mm, $a_{0}=0.3069\;\mathrm{mm},$ $a_{1}=0.5831\;\mathrm{mm},$ $a_{2}=1.1969\;\mathrm{mm},$ $a_{3}=1.4731\;\mathrm{mm},$ L=2.6516 mm, w=2.1483 mm, $R_{1}=0.008,\;R_{2}=0.018$. The material parameters of GdCOB with the cut of $(zxw)-30^{\circ }$ [19] have been obtained. For GdCOB crystals, Q = 10^3^ is utilized in the performed computations considering damping from material, air, and mounting.

**Figure 2:**
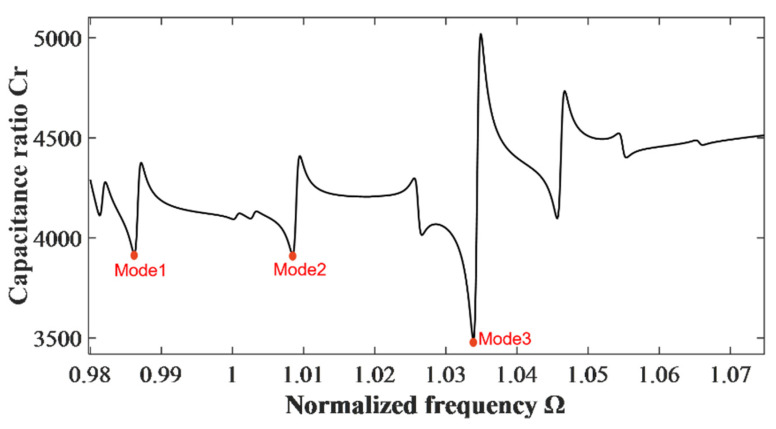
Capacitance ratio versus driving frequency. The three red dots (Modes 1–3) indicate the three resonance frequency points selected, namely 0.9862 *ω*_0_, 1.0085 *ω*_0_, and 1.0339 *ω*_0_.

**Figure 3:**
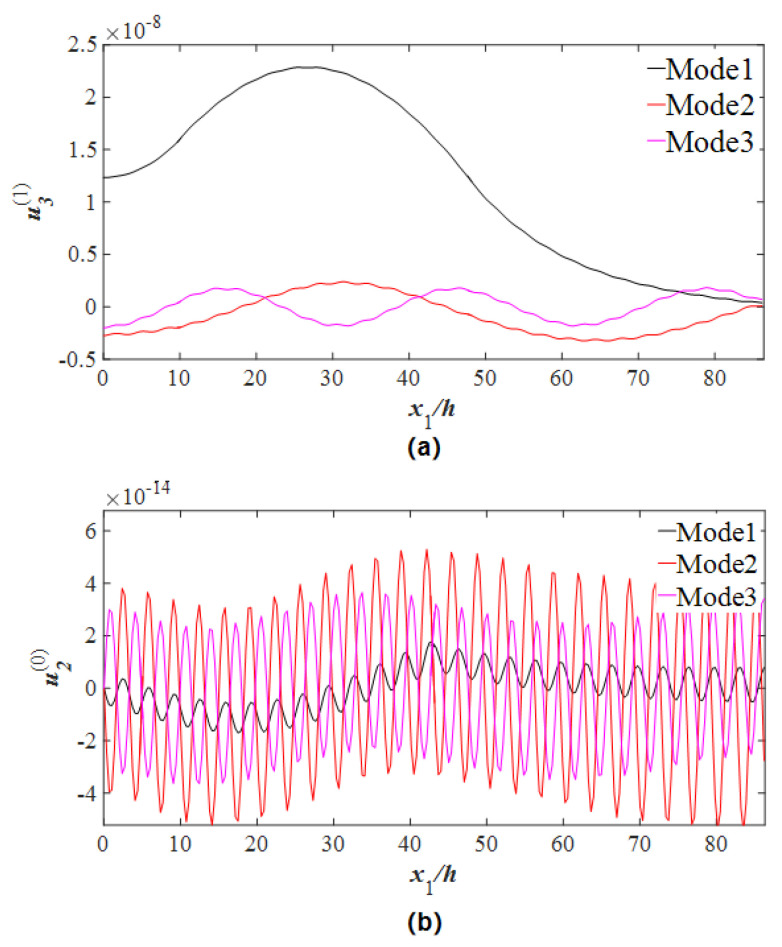
Strain distribution curves of the right half of the crystal plate on three modes. (**a**) Thickness-twist strain distribution. (**b**) Bending strain distribution.

Figure 2 shows the calculated curve of the capacitance ratio vs. normalization frequency. Resonance capacitance C is normalized by $C_{0}=4\varepsilon_{11}hp/(2c),$ namely capacitance ratio $C_{r}$ is obtained. $\omega_{0}$ represents the main frequency of the thickness-twist mode of an un-electrode plate and is utilized as a normalizing frequency, which is calculated by $\omega_{0}=(\pi /2h)\sqrt{c_{66}/\rho }$. Three main resonance frequencies Modes 1–3 in Figure 2 can be observed, namely 0.9862 $\omega_{0}$, 1.0085 $\omega_{0}$, and 1.0339 $\omega_{0}$, respectively. In order to find the mode with the best energy-trapping characteristics, it is necessary to plot the strain distributions of the thickness-twist mode $u_{3}^{\left(1\right)}(x_{1})$ and bending mode $u_{2}^{\left(0\right)}(x_{1})$ for three frequency points. The vibration characteristics for each frequency value are then analyzed. Figure 3a shows the thickness-twist mode (TT_1_) strain diagram, in which the TT_1_ strain amplitude corresponding to Mode 1 is large and concentrated in the electrode area, the vibration in the area uncovered by the electrode decays rapidly, and the vibration at the boundary of the resonator tends to be 0, which is a good energy-trapping effect. While the TT_1_ strain amplitude of Mode 2 and Mode 3 is small, and there is a node for the vibrations in the electrode area, which is not good for the energy-trapping effect. From the strain distribution plots of the bending modes (F_1_) shown in Figure 3b, it can be found that the vibration amplitude of the F_1_ mode corresponding to Mode 1 is very weak, thus the influence of flexure vibrations on thickness-twist vibration is negligible. While the vibration amplitudes of bending modes corresponding to Mode 2 and Mode 3 are larger. Therefore, Mode 1 is ideal for device applications.

## 5. The Influences of Stepped Electrodes on the Energy-Trapping Effect of GdCOB LFE Devices

**Figure 4:**
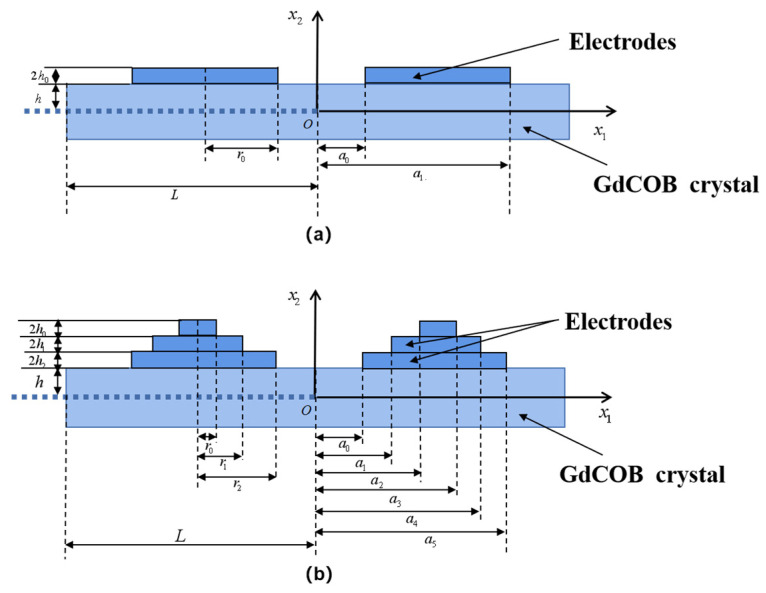
(**a**) GdCOB LFE device with single-step electrodes. (**b**) GdCOB LFE device with triple-step electrodes.

The effects of single- and triple-step electrodes on the energy-trapping effect of GdCOB LFE piezoelectric devices are considered and compared with those of the double-step electrodes. The device with double-step electrodes is shown in Figure 1, and those with single-step and triple-step electrodes are shown in Figure 4a,b, respectively. 

Here, only the region of $x_{1}>0$ is considered, and the crystal plate has a thickness of 2*h* and a length of 2*L*. The densities of the piezoelectric substrate material and the electrodes are ρ and $\rho^{\prime }$, respectively. Since the results in Section 4 show that for Mode 1 of GdCOB with the cut of $(zxw)-30^{\circ }$, the coupling between the TT_1_ mode and F_1_ mode is very weak, in the two-dimensional equations, we only consider the pure TT_1_ mode [24].

The governing equation is reduced to

(19) $$C_{55}u_{3,11}^{\left(1\right)}-\rho \omega_{\infty }^{2}u_{3}^{\left(1\right)}=\rho (1+3R){\ddot{u}}_{3}^{\left(1\right)},$$

where

(20) $$\omega_{\infty }^{2}=\frac{\pi^{2}}{4h^{2}}\frac{c44}{\rho },R(x)=\frac{2\rho^{\prime }h^{\prime }(x_{1})}{\rho h}.$$

For simple harmonic motion with frequency ω, the governing equation becomes

(21) $$C_{55}u_{3,11}^{\left(1\right)}-\rho [(1+3R)\omega^{2}-\omega_{\infty }^{2}]u_{3}^{\left(1\right)}=0.$$

### 5.1. Single-Step Electrodes

For the device with single-step electrodes, as shown in Figure 4a, the electrode thickness is satisfied

(22) $$2h(x)=\left\{ \begin{array}{l} 0,0<x_{1}<a_{0}\\ 2h_{0},a_{0}<x_{1}<a_{1}\\ 0,a_{1}<x_{1}<l \end{array}\right.$$

For the region of $x_{1}>0$, the corresponding governing equations and boundary conditions are as follows:

(23) $$\begin{array}{ll} u_{3,11}^{\left(1\right)}+\frac{\rho }{C_{55}}(\omega^{2}-\omega_{\infty }^{2})u_{3}^{\left(1\right)}=0, & (0<x_{1}<a_{0},a_{1}<x_{1}<l),\\ u_{3,11}^{\left(1\right)}+\frac{\rho }{C_{55}}[(1+3R_{0})(\omega^{2}-\omega_{0}^{2})]u_{3}^{\left(1\right)}=0, & (a_{1}<x_{1}<a_{2}). \end{array}$$

(24) $$\begin{array}{ll} u_{3,1}^{\left(1\right)}(0)=0, & u_{3}^{\left(1\right)}(l)=0,\\ u_{3}^{\left(1\right)}(a_{0}^{-})=u_{3}^{\left(1\right)}(a_{0}^{+}), & u_{3,1}^{\left(1\right)}(a_{0}^{-})=u_{3,1}^{\left(1\right)}(a_{0}^{+}),\\ u_{3}^{\left(1\right)}(a_{1}^{-})=u_{3}^{\left(1\right)}(a_{1}^{+}), & u_{3,1}^{\left(1\right)}(a_{1}^{-})=u_{3,1}^{\left(1\right)}(a_{1}^{+}), \end{array}$$

where

(25) $$\begin{array}{l} R_{0}=\frac{2\rho^{\prime }h_{0}}{\rho h},\\ \omega_{0}^{2}=\frac{\omega_{\infty }^{2}}{\left(1+3R_{0}\right)}<\omega_{\infty }^{2} \end{array}$$

when ω is in the range of $\left(\omega_{0},\omega_{\infty }\right)$, the vibrations of the device will be concentrated in the electrode region. Here, we consider the case for $\omega_{0}<\omega <\omega_{\infty }$. The corresponding motion governing equations are as follows:

(26) $$\begin{array}{l} u_{3,11}^{\left(1\right)}+\beta^{2}u_{3}^{\left(1\right)}=0,(0<x_{1}<a_{0},a_{3}<x_{1}<a_{4}),\\ u_{3,11}^{\left(1\right)}-\beta_{0}^{2}u_{3}^{\left(1\right)}=0,(a_{1}<x_{1}<a_{2}), \end{array}$$

where

(27) $$\begin{array}{l} \beta_{0}^{2}=\frac{\rho }{C55}(1+3R_{0})(\omega^{2}-\omega_{0}^{2})>0,\\ \beta^{2}=\frac{\rho }{C55}(\omega_{\infty }^{2}-\omega^{2})>0. \end{array}$$

The expression for the symmetric displacements can be written as follows:

(28) $$\begin{array}{ll} u_{3}^{\left(1\right)}=C_{0}\cos(\beta x_{1}), & 0<x_{1}<a_{0},\\ u_{3}^{\left(1\right)}=C_{1}\exp[-\beta_{0}(x_{1}-a_{0})], & a_{0}<x_{1}<a_{1},\\ u_{3}^{\left(1\right)}=C_{2}\cos[-\beta (x_{1}-a_{2})]+C_{2}^{\prime }\sin[-\beta (x_{1}-a_{2})] & a_{1}<x_{1}<a_{2}. \end{array}$$

Substituting Equation (29) into the corresponding boundary condition in Equation (24) yields

(29) $$\begin{array}{l} C_{0}\cos(\beta a_{0})=C_{1},\\ -\beta C_{0}\sin(\beta a_{0})=\beta_{1}C_{1},\\ C_{1}\exp(-\beta_{0}(a_{1}-a_{0}))=C_{2},\\ -\beta_{0}C_{1}\exp(-\beta_{0}(a_{1}-a_{0}))=-\beta C_{2}^{\prime }. \end{array}$$

The determinant of the coefficient matrix should be zero for nontrivial solutions, namely

(30) $$\left|\;\begin{array}{cccc} \cos(\beta a_{0}) & -1 & 0 & 0\\ -\beta sin(\beta a_{0}) & \beta_{0} & 0 & 0\\ 0 & \exp[-\beta_{0}(a_{1}-a_{0})] & -1 & 0\\ 0 & -\beta_{0}\exp[-\beta_{0}(a_{1}-a_{0})] & 0 & -\beta \end{array}\;\right|=0$$

### 5.2. Triple-Step Electrodes

For the device with triple-step electrodes, as shown in Figure 4b, the electrode thickness is satisfied

(31) $$2h(x)=\{\begin{array}{c} 0,0<x_{1}<a_{0}\\ 2h_{2},a_{0}<x_{1}<a_{1}\\ \begin{array}{l} 2h_{1},a_{1}<x_{1}<a_{2}\\ 2h_{0},a_{2}<x_{1}<a_{3} \end{array}\\ \begin{array}{l} 2h_{1},a_{3}<x_{1}<a_{4}\\ 2h_{2},a_{4}<x_{1}<a_{5} \end{array}\\ 0,a_{5}<x_{1}<l \end{array}$$

For the region of $x_{1}>0$, the corresponding governing equations and boundary conditions are

(32) $$\begin{array}{ll} u_{3,11}^{\left(1\right)}+\frac{\rho }{C_{55}}(\omega^{2}-\omega_{\infty }^{2})u_{3}^{\left(1\right)}=0, & \left(0<x_{1}<a_{0},a_{5}<x_{1}<l\right)\\ u_{3,11}^{\left(1\right)}+\frac{\rho }{C_{55}}[(1+3R_{2})(\omega^{2}-\omega_{2}^{2})]u_{3}^{\left(1\right)}=0, & \left(a_{0}<x_{1}<a_{1},a_{4}<x_{1}<a_{5}\right)\\ u_{3,11}^{\left(1\right)}+\frac{\rho }{C_{55}}[(1+3R_{1})(\omega^{2}-\omega_{1}^{2})]u_{3}^{\left(1\right)}=0, & \left(a_{1}<x_{1}<a_{2},a_{3}<x_{1}<a_{4}\right)\\ u_{3,11}^{\left(1\right)}+\frac{\rho }{C_{55}}[(1+3R_{0})(\omega^{2}-\omega_{0}^{2})]u_{3}^{\left(1\right)}=0, & \left(a_{2}<x_{1}<a_{3}\right) \end{array}$$

(33) $$\begin{array}{ll} u_{3,1}^{\left(1\right)}(0)=0, & u_{3}^{\left(1\right)}(l)=0\\ u_{3}^{\left(1\right)}(a_{0}^{-})=u_{3}^{\left(1\right)}(a_{0}^{+}), & u_{3,1}^{\left(1\right)}(a_{0}^{-})=u_{3,1}^{\left(1\right)}(a_{0}^{+}),\\ u_{3}^{\left(1\right)}(a_{1}^{-})=u_{3}^{\left(1\right)}(a_{1}^{+}), & u_{3,1}^{\left(1\right)}(a_{1}^{-})=u_{3,1}^{\left(1\right)}(a_{1}^{+}),\\ u_{3}^{\left(1\right)}(a_{2}^{-})=u_{3}^{\left(1\right)}(a_{2}^{+}), & u_{3,1}^{\left(1\right)}(a_{2}^{-})=u_{3,1}^{\left(1\right)}(a_{2}^{+}),\\ u_{3}^{\left(1\right)}(a_{3}^{-})=u_{3}^{\left(1\right)}(a_{3}^{+}), & u_{3,1}^{\left(1\right)}(a_{3}^{-})=u_{3,1}^{\left(1\right)}(a_{3}^{+}),\\ u_{3}^{\left(1\right)}(a_{4}^{-})=u_{3}^{\left(1\right)}(a_{4}^{+}), & u_{3,1}^{\left(1\right)}(a_{4}^{-})=u_{3,1}^{\left(1\right)}(a_{4}^{+}),\\ u_{3}^{\left(1\right)}(a_{5}^{-})=u_{3}^{\left(1\right)}(a_{5}^{+}), & u_{3,1}^{\left(1\right)}(a_{5}^{-})=u_{3,1}^{\left(1\right)}(a_{5}^{+}), \end{array}$$

where

(34) $$\begin{array}{l} R_{0}=\frac{2\rho^{\prime }h_{0}}{\rho h}>R_{1}=\frac{2\rho^{\prime }h_{1}}{\rho h}>R_{2}=\frac{2\rho^{\prime }h_{2}}{\rho h},\\ \omega_{0}^{2}=\frac{\omega_{\infty }^{2}}{\left(1+3R_{0}\right)}<\omega_{1}^{2}=\frac{\omega_{\infty }^{2}}{\left(1+3R_{1}\right)}<\omega_{2}^{2}=\frac{\omega_{\infty }^{2}}{\left(1+3R_{2}\right)}<\omega_{\infty }^{2}. \end{array}$$

when ω is at the range $\left(\omega_{0},\omega_{\infty }\right)$, the vibrations of the device are concentrated in the electrode region. Here, we consider the case for $\omega_{0}<\omega <\omega_{\infty }$.

The corresponding motion governing equations are as follows:

(35) $$\begin{array}{l} u_{3,11}^{\left(1\right)}-\beta_{0}^{2}u_{3}^{\left(1\right)}=0,(a_{2}<x_{1}<a_{3}),\\ u_{3,11}^{\left(1\right)}+\beta_{2}^{2}u_{3}^{\left(1\right)}=0,(a_{0}<x_{1}<a_{1},a_{4}<x_{1}<a_{5})\\ u_{3,11}^{\left(1\right)}+\beta_{1}^{2}u_{3}^{\left(1\right)}=0,(a_{1}<x_{1}<a_{2},a_{3}<x_{1}<a_{4})\\ u_{3,11}^{\left(1\right)}+\beta^{2}u_{3}^{\left(1\right)}=0,(0<x_{1}<a_{0},a_{5}<x_{1}<l) \end{array}$$

where

(36) $$\begin{array}{l} \beta_{0}^{2}=\frac{\rho }{C55}(1+3R_{0})(\omega^{2}-\omega_{0}^{2})>0,\\ \beta_{1}^{2}=\frac{\rho }{C55}(1+3R_{1})(\omega^{2}-\omega_{1}^{2})>0,\\ \beta_{2}^{2}=\frac{\rho }{C55}(1+3R_{2})(\omega^{2}-\omega_{2}^{2})>0,\\ \beta^{2}=\frac{\rho }{C55}(\omega_{\infty }^{2}-\omega^{2})>0. \end{array}$$

The expression for the symmetric displacements can be written as

(37) $$\begin{array}{l} u_{3}^{\left(1\right)}=C_{0}\cos(\beta x_{1}),0<x_{1}<a_{0},\\ u_{3}^{\left(1\right)}=C_{1}\cos[\beta_{2}(x_{1}-a_{0})]+C_{1}^{\prime }\sin[\beta_{2}(x_{1}-a_{0})],a_{0}<x_{1}<a_{1},\\ u_{3}^{\left(1\right)}=C_{2}\cos[\beta_{1}(x_{1}-a_{1})]+C_{2}^{\prime }\sin[\beta_{1}(x_{1}-a_{1})],a_{1}<x_{1}<a_{2},\\ u_{3}^{\left(1\right)}=C_{3}\exp[-\beta_{0}(x_{1}-a_{2})],a_{2}<x_{1}<a_{3},\\ u_{3}^{\left(1\right)}=C_{4}\cos[\beta_{1}(x_{1}-a_{3})]+C_{4}^{\prime }\sin[\beta_{1}(x_{1}-a_{3})],a_{3}<x_{1}<a_{4},\\ u_{3}^{\left(1\right)}=C_{5}\cos[\beta_{2}(x_{1}-a_{4})]+C_{5}^{\prime }\sin[\beta_{2}(x_{1}-a_{4})],a_{4}<x_{1}<a_{5},\\ u_{3}^{\left(1\right)}=C_{6}\exp[-\beta (x_{1}-a_{5})]+C_{6}^{\prime }{\begin{array}{cc} \exp[-\beta (x_{1}-a_{5})], & a \end{array}}_{5}<x_{1}<l. \end{array}$$

Substituting Equation (37) into the corresponding boundary condition Equation (33) yields

(38) $$\begin{array}{l} C_{0}\cos(\beta a_{0})=C_{1},\\ -\beta C_{0}\sin(\beta a_{0})=\beta_{2}C_{1}^{\prime },\\ C_{1}\cos[\beta_{2}(a_{1}-a_{0})]+C_{1}^{\prime }\sin[\beta_{2}(a_{1}-a_{0})]=C_{2},\\ -\beta_{2}C_{1}\sin[\beta_{2}(a_{1}-a_{0})]+\beta_{2}C_{1}^{\prime }\cos[\beta_{2}(a_{1}-a_{0})]=\beta_{1}C_{2}^{\prime },\\ C_{2}\cos[\beta_{1}(a_{2}-a_{1})]+C_{2}^{\prime }\sin[\beta_{1}(a_{2}-a_{1})]=C_{3},\\ -\beta_{1}C_{2}\sin[\beta_{1}(a_{2}-a_{1})]+\beta_{1}C_{2}^{\prime }\cos[\beta_{1}(a_{2}-a_{1})]=-\beta_{0}C_{3},\\ C_{3}\exp(-\beta_{0}(a_{3}-a_{2}))=C_{4},\\ -\beta_{0}C_{3}\exp(-\beta_{0}(a_{3}-a_{2}))=\beta_{1}C_{4}^{\prime },\\ C_{4}\cos[\beta_{1}(a_{4}-a_{3})]+C_{4}^{\prime }\sin[\beta_{1}(a_{4}-a_{3})]=C_{5},\\ -\beta_{1}C_{4}\sin[\beta_{1}(a_{4}-a_{3})]+\beta_{1}C_{4}^{\prime }\cos[\beta_{1}(a_{4}-a_{3})]=\beta_{2}C_{5}^{\prime },\\ C_{5}\cos[\beta_{2}(a_{5}-a_{4})]+C_{5}^{\prime }\sin[\beta_{2}(a_{5}-a_{4})]=C_{6},\\ -\beta_{2}C_{5}\sin[\beta_{2}(a_{5}-a_{4})]+\beta_{2}C_{5}^{\prime }\cos[\beta_{2}(a_{5}-a_{4})]=-\beta C_{6}-\beta C_{6}^{\prime }, \end{array}$$

The determinant of the coefficient matrix should be zero for nontrivial solutions, namely

(39) $$\begin{array}{l} |\begin{array}{cccccc} \cos(\beta a_{0}) & -1 & 0 & 0 & 0 & 0\\ -\beta sin(\beta a_{0}) & 0 & -\beta_{2} & 0 & 0 & 0\\ 0 & \cos[\beta_{2}(a_{1}-a_{0})] & \sin[\beta_{2}(a_{1}-a_{0})] & -1 & 0 & 0\\ 0 & -\beta_{2}\sin[\beta_{2}(a_{1}-a_{0})] & \beta_{2}\cos[\beta_{2}(a_{1}-a_{0})] & 0 & -\beta_{1} & 0\\ 0 & 0 & 0 & \cos[\beta_{1}(a_{2}-a_{1})] & \sin[\beta_{2}(a_{1}-a_{0})] & -1\\ 0 & 0 & 0 & -\beta_{1}\sin[\beta_{1}(a_{2}-a_{1})] & -\beta_{1}\sin[\beta_{1}(a_{2}-a_{1})] & \beta_{0}\\ 0 & 0 & 0 & 0 & 0 & \exp[-\beta_{0}(a_{3}-a_{2})]\\ 0 & 0 & 0 & 0 & 0 & \beta_{0}\exp[-\beta_{0}(a_{3}-a_{2})]\\ 0 & 0 & 0 & 0 & 0 & 0\\ 0 & 0 & 0 & 0 & 0 & 0\\ 0 & 0 & 0 & 0 & 0 & 0\\ 0 & 0 & 0 & 0 & 0 & 0 \end{array}\\ \begin{array}{cccccc} 0 & 0 & 0 & 0 & 0 & 0\\ 0 & 0 & 0 & 0 & 0 & 0\\ 0 & 0 & 0 & 0 & 0 & 0\\ 0 & 0 & 0 & 0 & 0 & 0\\ 0 & 0 & 0 & 0 & 0 & 0\\ 0 & 0 & 0 & 0 & 0 & 0\\ -1 & 0 & 0 & 0 & 0 & 0\\ 0 & -\beta_{1} & 0 & 0 & 0 & 0\\ \cos[\beta_{1}(a_{4}-a_{3})] & \sin[\beta_{1}(a_{4}-a_{3})] & -1 & 0 & 0 & 0\\ -\beta_{1}\sin[\beta_{1}(a_{4}-a_{3})] & \beta_{1}\cos[\beta_{1}(a_{4}-a_{3})] & 0 & -\beta_{2} & 0 & 0\\ 0 & 0 & \cos[\beta_{2}(a_{5}-a_{4})] & \sin[\beta_{2}(a_{5}-a_{4})] & -1 & 0\\ 0 & 0 & -\beta_{2}\sin[\beta_{2}(a_{5}-a_{4})] & \beta_{2}\cos[\beta_{2}(a_{5}-a_{4})] & \beta & \beta \end{array}|=0 \end{array}$$

Au is selected as the electrode material of the device, and the parameters of the GdCOB LFE bulk acoustic wave device are set as follows:

(40) $$\begin{array}{l} \rho =3819\;\mathrm{kg}/m^{3},\;c_{44}=55.87\times 10^{9}\;N/m^{2},\;a_{0}=0.3069\;\mathrm{mm},\;a_{1}=0.5831\;\mathrm{mm},\;\\ a_{2}=1.1969\;\mathrm{mm},\;a_{3}=1.4731\;\mathrm{mm},\;L=2.6516\;\mathrm{mm},\rho^{\prime }=19300\;\mathrm{kg}/m^{3},h=0.0614\;\mathrm{mm} \end{array}$$

**Figure 5:**
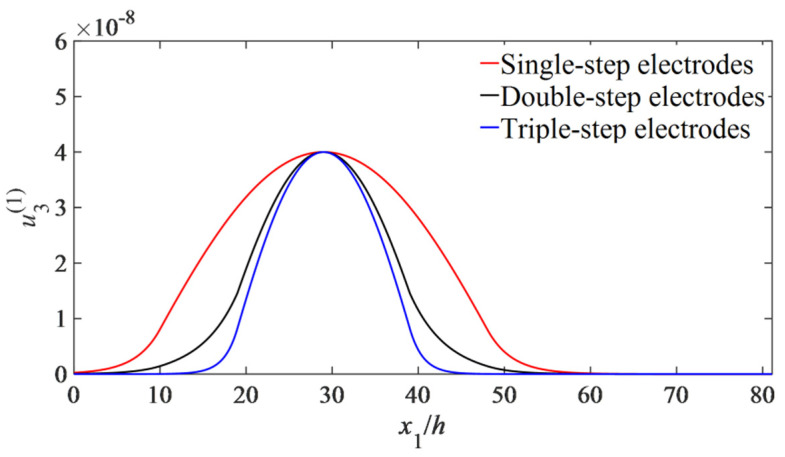
Influences of the electrode type on the energy trapping of the GdCOB LFE device. Strain distribution of the three-layer stepped electrode indicated by the blue line in the figure is more centralized compared with those of the other two types.

To evaluate the energy-trapping effect, strain distributions in the $x_{1}$ direction are calculated by Equations (37)–(39), and a comparison of the energy-trapping effect is shown in Figure 5, from which it can be seen that for triple-step electrodes, more strain energy is more centralized compared with those of the other two types; thus, its energy-trapping effect is better than others. 

**Figure 6:**
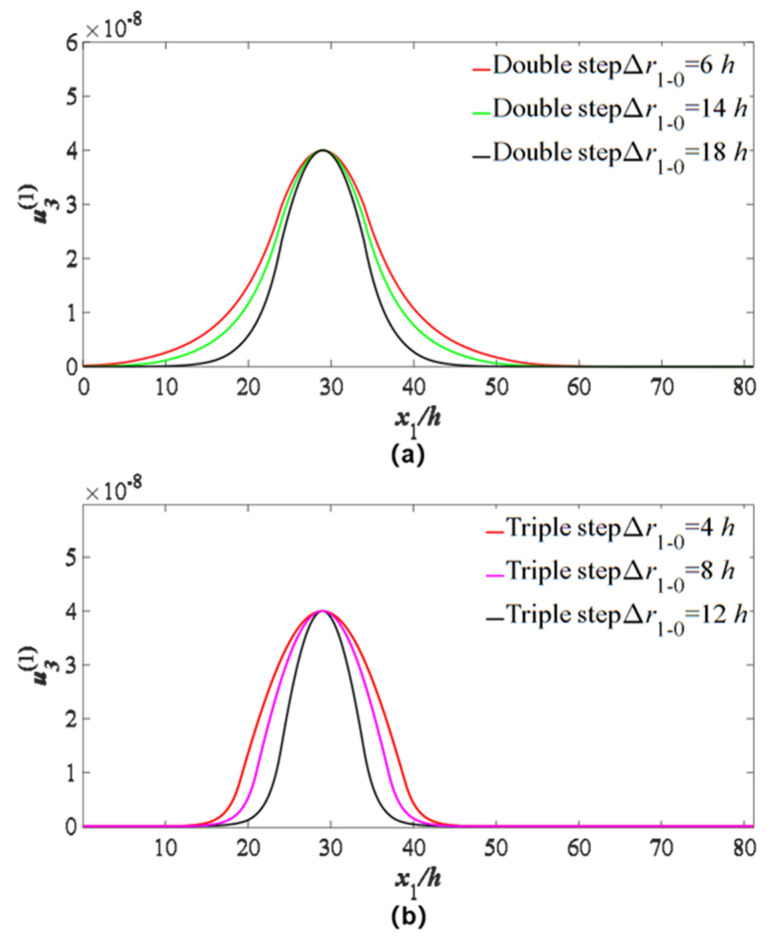

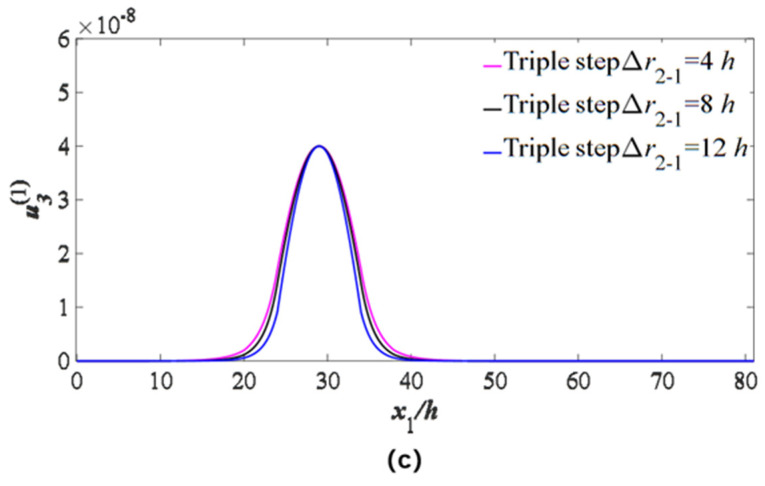
Influences of electrode radius difference on TT_1_ strain distribution $u_{3}^{\left(1\right)}$. (**a**) Double-step electrodes: only the radius of the upper electrode is changed, and the radius of the lower electrode is kept for 24 *h*. (**b**) Three-layer stepped electrodes: the radius of the middle and bottom electrodes is kept for 18 *h* and 24 *h*, respectively. Only the radius of the upper electrode is changed. (**c**) Three-layer stepped electrodes: the radius of the upper and bottom electrodes is kept for 8 *h* and 24 *h*, respectively. Only the radius of the middle electrode is changed.

**Figure 7:**
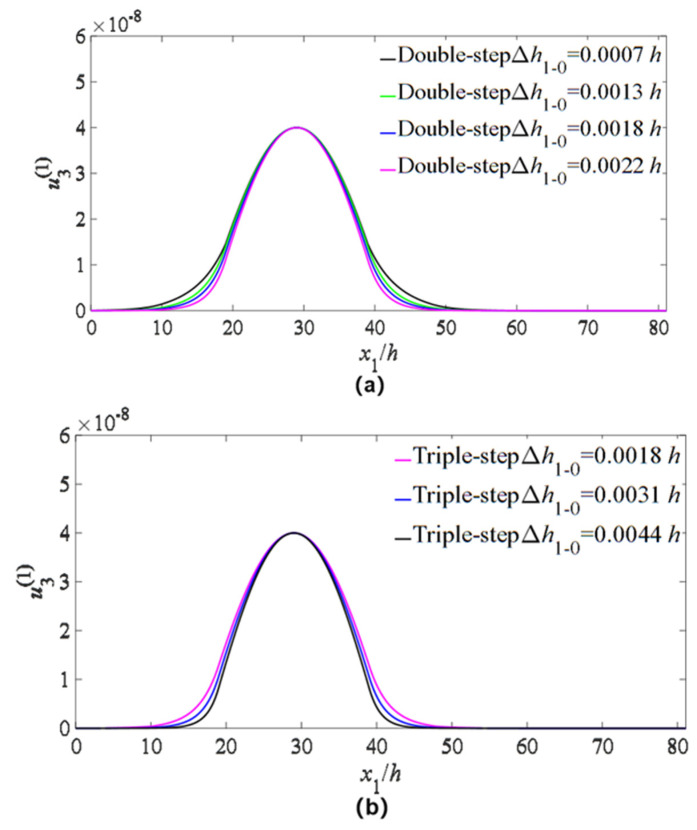

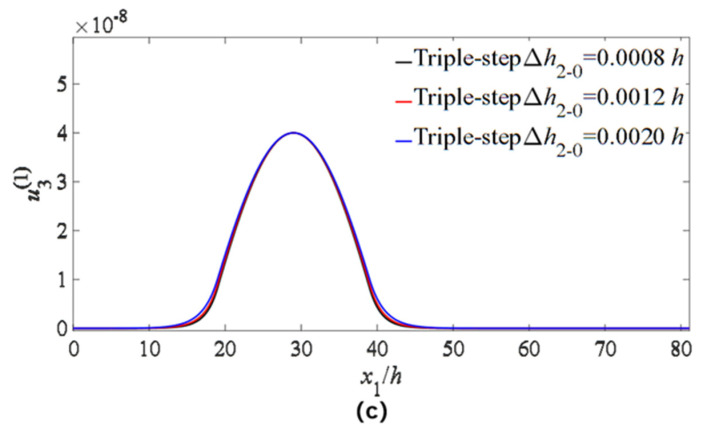
Influences of electrode thickness difference on TT_1_ strain distribution $u_{3}^{\left(1\right)}$. (**a**) Double-step electrodes: only the thickness of the upper electrode is changed, and the radius of the lower electrode is kept for 0.008 *h*. (**b**) Three-layer stepped electrodes: the thickness of the middle and bottom electrodes is kept for 0.0015 *h* and 0.0008 *h*, respectively, and only the thickness of the upper electrode is changed. (**c**) Three-layer stepped electrodes: the thickness of the upper and middle electrodes is kept for 0.0026 *h* and 0.0015 *h*, respectively, and only the thickness of the bottom electrode is changed.

In order to check the influences of electrode parameters on the energy trapping of the device, the changes of a single electrode parameter (electrode radius difference and electrode thickness difference) are introduced, and the vibration distributions of the main mode are plotted. The results are shown in Figure 6 (electrode radius difference) and Figure 7 (electrode thickness). 

For the case of double-step electrodes, only the radius of the upper electrode is changed, and the radius of the lower electrode is kept for 24 *h*. The Δr1−0 represents the radius difference between the lower and upper electrodes. From Figure 6a, it is shown that when the radius difference increases, a better energy-trapping effect can be obtained. For the case of triple-step electrodes, firstly, the radius of the middle and bottom electrodes is kept for 18 *h* and 24 *h*, respectively. Only the radius of the upper electrode is changed, and the Δr1−0 represents the radius difference between the middle and upper electrodes. The results are shown in Figure 6b, from which it is shown that when Δr1−0 is smaller, the energy-trapping effect is better. Secondly, the radius of the upper and bottom electrodes is kept for 8 *h* and 24 *h*, respectively. Only the radius of the middle electrode is changed, and the Δr2−1 represents the radius difference between the bottom and middle electrodes. The results are shown in Figure 6c, from which it is also shown that a larger radius difference leads to a better energy-trapping effect.

For the case of double-step electrodes, only the thickness of the upper electrode is changed, and the radius of the lower electrode is kept for 0.008 *h*. The Δh1−0 represents the thickness difference between the lower and upper electrodes. From Figure 7a, it is shown that when the thickness difference increases, a better energy-trapping effect can be obtained. In the case of triple-step electrodes, Firstly, the radius of the middle and bottom electrodes is kept for 0.0015 *h* and 0.0008 *h*, respectively. Only the radius of the upper electrode is changed, and the Δh1−0 represents the radius difference between the middle and upper electrodes. The results are shown in Figure 7b, from which it is also shown that a larger radius difference leads to a better energy-trapping effect. The radius of the upper and middle electrodes is kept for 0.0026 *h* and 0.0015 *h*, respectively. Only the radius of the bottom electrode is changed, and the Δr2−1 represents the radius difference between the bottom and middle electrodes. The results are shown in Figure 6c, and it can be seen that the thicker the electrodes are, the better the energy-trapping effect is; however, the effect is relatively weaker compared to Figure 7b. Therefore, for the step-electrode LFE device, changing the radius difference and thickness difference can lead to a better energy-trapping effect of the device. 

## 6. Conclusions

In this paper, high-frequency forced vibrations of the LFE device with stepped electrodes based on monoclinic GdCOB crystals are investigated. The dynamic capacitance ratio is calculated, and the influences of the number, size, and thickness of stepped electrodes on the energy-trapping effect of the device are analyzed. The results show that the number of electrode layers has an obvious influence on the energy-trapping effect of the device; namely, with an increase in the number of electrode layers, the energy-trapping effect of the device becomes stronger. With the increase in electrode thickness difference, the corresponding device energy-trapping effect becomes stronger. With the increase in the electrode radius difference, the energy-trapping effect of the device is gradually enhanced. The results of this paper can provide a reliable theoretical basis for the parameter design of LFE devices with stepped electrodes for good energy-trapping effects.

## Data Availability

Data are contained within the article.
